# A Transdiagnostic, Emotion Regulation App (Eda) for Children: Design, Development, and Lessons Learned

**DOI:** 10.2196/28300

**Published:** 2022-01-19

**Authors:** Bettina Moltrecht, Praveetha Patalay, Holly Alice Bear, Jessica Deighton, Julian Edbrooke-Childs

**Affiliations:** 1 Evidence-based practice unit Department of Clinical, Educational and Health Psychology University College London London United Kingdom; 2 Centre for Longitudinal Studies Institute of Education University College London London United Kingdom; 3 Department of Psychiatry University of Oxford Oxford United Kingdom

**Keywords:** mHealth, participatory design, emotion regulation, interdisciplinary development, child mental health

## Abstract

**Background:**

Digital interventions, including mobile apps, represent a promising means of providing effective mental health support to children and young people. Despite the increased availability of mental health apps, there is a significant gap for this age group, especially for children (aged 10-12 years). Research investigating the effectiveness and development process of child mental health apps is limited, and the field faces persistent issues in relation to low user uptake and engagement, which is assumed to be a result of limited user involvement in the design process.

**Objective:**

This study aims to present the development and design process of a new mental health app for children that targets their emotion regulation abilities. We describe the creation of a new interdisciplinary development framework to guide the design process and explain how each activity informed different app features.

**Methods:**

The first 2 stages of the framework used a variety of methods, including weekly classroom observations over a 6-month period (20 in total); public engagement events with the target group (N=21); synthesis of the existing evidence as part of a meta-analysis; a series of co-design and participatory workshops with young users (N=33), clinicians (N=7), researchers (N=12), app developers (N=1), and designers (N=2); and finally, testing of the first high-tech prototype (N=15).

**Results:**

For the interdisciplinary framework, we drew on methods derived from the Medical Research Council framework for complex interventions, the patient–clinician framework, and the Druin cooperative inquiry. The classroom observations, public engagement events, and synthesis of the existing evidence informed the first key pillars of the app and wireframes. Subsequently, a series of workshops shaped and reshaped the content and app features, including games, psychoeducational films, and practice modules. On the basis of the prototype testing sessions, we made further adjustments to improve the app.

**Conclusions:**

Although mobile apps could be highly suitable to support children’s mental health on a wider scale, there is little guidance on how these interventions could be designed and developed. The involvement of young users across different design activities is very valuable. We hope that our interdisciplinary framework and description of the used methods will be helpful to others who are hoping to develop mental health apps for children and young people.

## Introduction

### Background

It has been estimated that approximately 10%-20% of children and young people worldwide experience mental health problems, making it one of the leading causes of disability in this population [1,2]. Addressing the rising number of mental health problems in young people is a major public health concern. International studies indicate that >60% of young people do not have access to adequate (or any) treatment [3], highlighting the urgent need for innovative approaches to tackling this problem. Mental health apps present a promising means of addressing this challenge by making mental health support more widely accessible to young people. Our research team developed a new mental health app that aims to support children (aged 10-12 years) by enhancing their emotion regulation skills. Difficulties with emotion regulation are seen in a wide range of mental health problems, and recent evidence suggests that enhancing emotion regulation in children and adolescents is related to improvements in mental health regardless of the type of disorder or intervention [4]. To date, guidelines and studies on the development of mental health apps for children are lacking. This study aims to fill this gap.

### Digital Mental Health Landscape for Youth

Although it has been suggested that digital mental health interventions can be efficacious in both preventing and treating mental health problems in young people (aged ≥12 years) [5-7], recent systematic reviews found that in comparison to the adult literature, research investigating the effectiveness of digital interventions for children and young people is lagging [7,8]. Only a few mental health apps have been designed and tested specifically for young people (aged ≥12 years); however, even fewer are available for children aged <12 years. The latest systematic reviews identified only 2 mental health apps for children, thereby highlighting the significant evidence gap and limited availability of suitable digital interventions for this age group [7,9]. Despite these considerable limitations, digital interventions are expected to be highly accepted by young people because of the high degree of anonymity they provide. Furthermore, they are cost‐effective and, if designed appropriately, are highly applicable across different contexts [10].

Most available mental health apps have low uptake and engagement levels [11,12], which are considered essential to securing their effectiveness [13,14]. Various methods have been suggested to increase engagement levels with digital interventions, including the involvement of users in the development and design process as part of user-centered design methods. A recent review of 30 studies and another meta-review of 21 studies demonstrated that most digital mental health interventions, which targeted children and young people, neglected the use of such methods, which is reflected in the highly uniform design across these interventions, where psychoeducation often represents the main intervention component [15,16]. In terms of specific mental health apps for children (aged <12 years), we were unable to identify any app that involved target users during the development and design stages, thereby further emphasizing the importance of this paper.

A closer look at the digital mental health landscape indicates that most interventions draw on evidence-based treatments that target specific symptoms or diagnoses [17,18]. Initially, the recycling approach of taking existing interventions and transferring them to digital platforms helped the field to move forward quickly; however, this approach has increasingly been criticized as it provides little room for innovation and improvement [17]. Chandrashekar [19] summarized the key features of highly effective and engaging mental health apps and specifically highlighted components targeting transdiagnostic mechanisms. Transdiagnostic mechanisms are not specific to one disorder but are present across different mental health problems. The focus on transdiagnostic mechanisms has also enhanced traditional psychotherapeutic approaches in the past [20,21]. One such mechanism that has been repeatedly emphasized as a highly promising treatment and prevention target is emotion dysregulation.

### Emotion Regulation as an Intervention Target

Deficits in emotion regulation—or the ability to monitor, evaluate, and modify one’s emotional reactions to accomplish one’s goals [22]—have been identified as a risk and maintenance factor for mental ill health. Developmental research has demonstrated that higher emotion dysregulation in children is associated with greater mental health difficulties concurrently and later in life. Recent meta-analyses have indicated that interventions that effectively reduce emotion dysregulation in children also reduce psychopathological symptoms, irrespective of the intervention type or clinical diagnosis [4,23]. With respect to our target group, that is, children aged 10 to 12 years, the latter is of particular importance, as high comorbidity rates are common in this group and symptom presentations are often not clear-cut.

The transdiagnostic approach has also been deemed suitable for mental health prevention programs. Forbes et al [24] recently highlighted that targeting transdiagnostic factors in mental health prevention has the potential to activate a range of related, beneficial developmental cascades, such as social or academic development. Furthermore, they argued that transdiagnostic approaches reduce the burden on schools, for whom it is difficult to provide a multitude of short-lived programs, each trying to tackle a different problem [24].

To the best of our knowledge, there is currently no app intervention that targets emotion regulation as a transdiagnostic factor in late childhood (aged 10-12 years), although this period has been highlighted as a critical stage in achieving maximum impact in terms of youth mental health prevention [25]. Moreover, in the United Kingdom, late childhood (ages 10-12 years) is characterized by the transition from primary to secondary school, which is frequently experienced as stressful by children, thereby strengthening the case for an intervention that supports children before and during this transition period [26].

### This Paper’s Objectives

With respect to the existing limitations in the field, we present the development process of a new, transdiagnostic mental health app for children, which puts the young user group in the center of the design process. To achieve this, we have created a new development framework that draws on methodologies from the fields of psychology, human–computer interaction (HCI), and user design. In the following sections, we (1) describe the interdisciplinary design, development, and refinement process; (2) take the reader through the different stages and research activities; (3) describe the various app features and explain how they were informed by the research activities; and finally, (4) share important lessons that were learned and considerations for future activities.

### Formulation of Our Interdisciplinary Development Framework

#### Developing a Complex Digital Intervention

**Figure 1:**
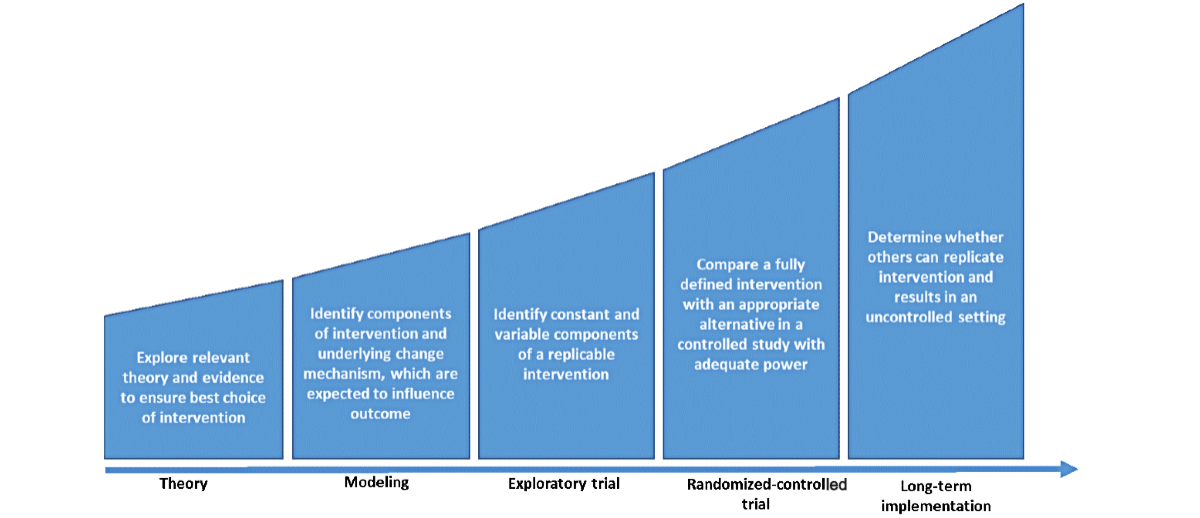
Medical Research Council framework for complex interventions.

**Figure 2:**
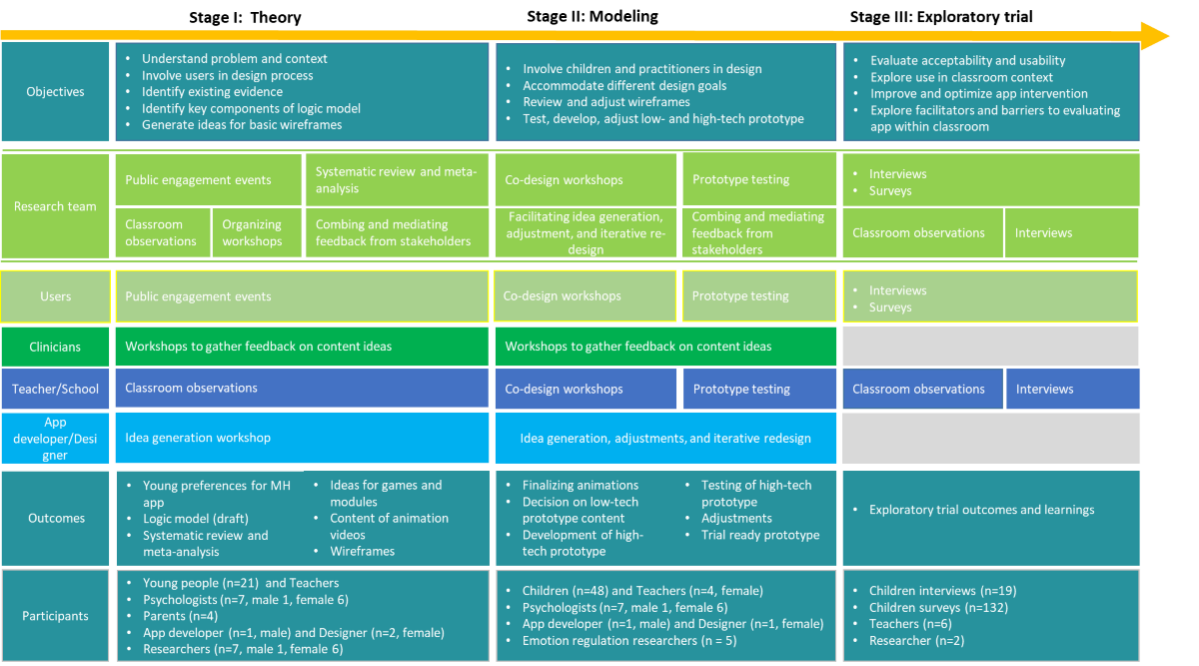
Development framework and research activity outline of the present app.

The present mental health app is considered a complex intervention as it involves multiple, interconnected, and interacting components [27,28]. In line with that, we used the first 3 stages of the Medical Research Council (Figure 1) framework for complex interventions to guide the development and evaluation process of the present mental health app. We first explored relevant theories and existing evidence to identify promising intervention components. In the next stage, the research team focused on identifying the underlying mechanisms that might influence the preferred outcome to incorporate them in the design of the intervention. This paper focuses primarily on the early development and design stages to address existing gaps in the literature. Therefore, we only present the research activities of the first 2 stages, as depicted in Figure 2. The findings of the third stage, the exploratory trial, are published elsewhere [29].

#### Designing a Complex Digital Intervention

Although the Medical Research Council framework provides valuable guidelines for the development and evaluation of complex interventions, it provides little information on the design of intervention components [30]. Hence, we drew on 2 frameworks rooted in the fields of HCI and user-centered design.

The patient–clinician–designer framework provides guidance on how to structure the design and content creation process of digital interventions for mental illness [31]. It aims to meet the complex requirements when designing user-centered interventions for mental illnesses by taking into account different perspectives (ie, patient vs clinician) and design goals. It describes how 5 key principles, based on user-centered design methodology, can be applied in the design process and divides it into four design phases: (1) understanding the illness and its challenges, (2) involving users in the design, (3) mediating co-design activities between users and professionals, and (4) accommodating different evaluation goals.

With respect to our target user group, that is, children at the end of primary school (aged 10-12 years), we decided to incorporate the Druin [32] *cooperative inquiry* framework, which provides specific techniques for involving young users in the design process of technologies and is widely used in the field. This framework highlights the importance of involving children as partners in the whole process instead of merely letting them test an almost finished prototype or end product. Druin [32] emphasizes the benefits of conducting fieldwork (ie, *contextual inquiry*) first, as it allows researchers to detect relevant contextual information, including patterns of activities, ways of communication, and other artifacts. In addition, it has been reported that discussing design features in the relevant context (eg, school and home) makes it easier for children to express ideas and provide suggestions [33]. Finally, the framework calls for the importance of visualizing ideas through low- and high-tech prototypes, as this offers children more concrete ways to elaborate on ideas and reject or refine them.

#### Present Development Framework

We combined the 3 frameworks outlined above, which allowed us to take a highly interdisciplinary approach (Figure 2). For each stage of the development process, we used a unique set of methodologies derived from different disciplines. The research team was involved in all the activities as a linking point and served as a mediator between different stakeholders.

The research team comprised 5 child and adolescent mental health researchers, of whom 2 have extensive experience in conducting digital health research (BM and JEC), 1 has a background in clinical psychology (BM), and 4 have extensive experience in designing, delivering, and evaluating school mental health programs (JD, JEC, PP, and HAB). A total of 3 authors (BM, JEC, and JD) are part of an international training network on technology-enabled mental health systems for young people, with experts from different disciplines (ie, computer sciences, psychology, medicine, data privacy, and design), who were consulted throughout the process. BM also has extensive training in applying HCI and user experience (UX) techniques. Figure 2 depicts the stakeholders involved at each stage, including app developers, clinicians, UX and graphic designers, young people, teachers, and parents.

## Methods

### Stage 1: Identifying Theory, Evidence, and Challenges

#### Objectives

As shown in Figure 2, this stage focuses on understanding the context and the problem at hand. We generate initial ideas with key stakeholders and test the feasibility of potential research activities for stage 2. Furthermore, we summarize the existing evidence to create the underlying logic model of the intervention, including its active ingredients and expected outcomes [34].

#### Activities and Data Analysis

In line with our framework, we conducted (1) multiple classroom observations, (2) a systematic review and meta-analysis to summarize the evidence for existing psychological interventions and their effectiveness in enhancing emotion regulation skills in youth, and (3) 2 consultation groups with young people. Notes and materials (ie, drawings, sticky notes, outcomes of exercises, and reflective notes) produced during (1) classroom observations and (2) consultation groups were analyzed using reflexive thematic analysis as described by Braun and Clarke [35,36]. BM manually coded the data, detailing inductive descriptive codes by highlighting and categorizing similar phrases, words, or patterns across the data. This was done in NVivo 11 (QSR International NVivo) or Microsoft Excel (further specified in the following sections). Themes were created using mind-mapping exercises and refined through discussions with all authors. Occasionally, we also involved app developers, designers, and clinicians, as specified below.

#### School Visits and Classroom Observations

Familiarization with the user and their environment as part of a fieldwork exercise is a central tenet of the cooperative inquiry framework. Clinical research has also shown that the identification of user resources within the intervention context is a significant determining factor of an interventions’ effectiveness [37].

Schools are considered key players in youth mental health provision [38,39]. We collaborated closely with 2 schools for this project, resulting in weekly school visits across a 6-month period, with a total of 20 observations. This allowed us to identify common challenges that children and teachers face, especially in relation to emotion dysregulation and resulting behavioral difficulties (eg, not being able to concentrate, disrupting the teaching process, and distracting other children). We became familiar with their everyday practices and issues related to the school setting and gained valuable insights into what children and teachers do to manage difficult emotions at school (Table 1).

**Table 1:** Outcomes and implications based on school observations.

Observations	Design implication and goals
Teachers and children use different devices, including tablets, PCs, and smartboards, during lessons.	Web-based app that can be accessed from different devices
Children try different strategies that help them in the classroom. Sometimes, these are agreed with the teacher.	Let children create a list of *tools*, which contains personal strategies and provides suggestions
Children struggle to draw on strategies when they have very intense emotions. Some teachers direct children in need to a quiet corner.	Add a function that gives quick access to guided strategy to provide in-the-moment support
Children are familiar with breathing exercises and time-outs.	Guided relaxation, breathing, and mindfulness exercises
Children report on certain situations in which they find it difficult to regulate their emotions and where this impedes their goals.	Integrate children’s stories as examples in content to make it more relevant to the target group
Teachers use a range of strategies, some that help specific individuals when needed and some that they apply to the whole class.	Design an intervention that can be used with the whole class, as well as for individual children

The lead author (BM) was able to observe different lessons, classes, and teachers in both schools. BM took notes during the observations and reflective notes afterward. BM was also able to ask teachers about their understanding of emotion dysregulation, its role in the classroom, and how children and teachers managed situations where children experienced intense feelings. During the school visits, teachers and children most frequently mentioned 1 strategy to manage emotion dysregulation in the classroom: the use of quiet corners or so-called *time-out zones*.

Time-out zones are defined areas in the classroom where students are directed when they show difficult behavior, struggle to concentrate, or distract other pupils in class. Some classrooms were divided into different zones, representing different types of support (eg, zones closer to the front to facilitate concentration and zones closer to the back for time-outs). The time-out zones often included a sofa or pillows to sit on, and children had access to books and other tools to help them calm down. On the basis of this observation, storing a tablet with the app intervention in the time-out zone seemed to be a suitable approach to implement the app in the classroom context.

Children reported that they themselves or together with a teacher had identified strategies to manage difficult feelings, such as playing with putty, stepping outside, reading a book, or listening to music in a quiet corner. This list of emotion regulation strategies inspired the implementation of the digital toolbox in the app see *Intervention Description* below).

Although insights from the school visits significantly influenced some design concepts in the app, the collaboration with the schools also helped us access parents and other professionals who we were able to consult on parent–teacher days about the app.

#### Public Consultation Groups

A total of 2 consultation groups were conducted as part of a patient and public involvement (PPI) event. Participants were recruited through the center’s network and existing collaborations with other third-sector child mental health organizations. Organization leads contacted young people or their parents who previously consented to be contacted for PPI events.

The PPI events involved 21 *young research advisors* aged between 12 and 19 years and had an even distribution of female and male participants. The term *young research advisor* is a special term that is used to describe a group of young people who have been service users themselves and received specific training that prepares them to work with researchers. The research team worked with this specific group, despite them being slightly older than the target group, because of their prior training and experience of working with researchers. This had a number of benefits. They were familiar with common research processes and had an existing relationship with the workshop facilitators, which secured good engagement. This allowed us to ask questions that were more complex and receive direct feedback on specific workshop activities.

The young advisors were reimbursed for their time in line with the organization’s internal arrangements. The lead author and 2 PPI leaders who were familiar with young advisors facilitated the workshops.

1. Each PPI event included an icebreaker exercise, an introduction to the topic (eg, mental health and digital interventions), and a discussion of the following questions:
2. What is mental health for you, and how do you take care of it?
3. How can technology support young people’s mental health or emotion regulation?
4. What are young people’s perceived barriers to and facilitators of the use of mental health apps?
5. How can research involve children and young people in the design process of mental health technology?

For some exercises, the groups were split into smaller teams first, where they brainstormed together and collected ideas on big sheets of paper. Subsequently, each group presented their ideas and discussed them with a larger group. BM was present at each PPI event to observe participants, ask follow-up questions, and take notes. Materials produced as part of the PPI groups (ie, drawings and notes resulting from exercises) and written notes by the lead author were thematically analyzed [35,36]. On the basis of the identified themes, a list of *do’s and don’ts* for mental health apps was developed (Table 2). If the young advisors had suggested possible solutions in the workshop, they were included; however, the research team also consulted the app developer and graphic designer afterward to identify possible solutions (marked with *a* in Table 2).

**Table 2:** Identified *do’s* and *don’ts* for mental health apps based on patient and public involvement group.

	Please do	Please avoid	App solution
**Accessibility**			
	Available across devices Affordable for a young person Available offline	Advertisement In-app purchases Too much data or Wi-Fi	Web-based app^a^ No costs Data or Wi-Fi for first-time log-in and updates^a^
**Engagement**			
	Interactive, games, and tracking Social connection and community Make use of users’ feedback and provide relevant updates	Push notifications Dead website or app Information or text only	Selection of games Digital agent for interaction Multimedia content Feedback option in the app
**Design**			
	Customizable features Age appropriate (language and design) Intuitive and easy to use	Childish Clunky Text only	Customizable features Designed and tested by users
**Data and technology**			
	Use cloud service to limit storage space Transparent data tracking User control over data or tracking Data security and privacy	Requires too much data Crashes or is slow Hidden data tracking	Google Analytics provides insight for general use of app content^a^ No individual data tracking through app^a^
**Mental health–specific**			
	Teach and educate Increase understanding Opportunity to practice Facilitate social connectedness Signposting to services	Signposting only Text only	Content that educates and increases understanding Practice modules Digital agent to feel socially connected Signposting

^a^Suggested by the app developer.

#### Considerations and Design Implications

The patient–clinician–designer and cooperative inquiry framework recommend the inclusion of target users directly into the design process. We explored the usability of potential co-design methods with this group before using them in workshops with younger, untrained children. However, the PPI participants were significantly older (aged 12-19 years) than the expected user group, which might explain some of the difficulties that we faced when working with younger children in the co-design workshops (see *Co-design Workshops* section).

#### Systematic Review and Meta-analysis

We conducted a systematic review and meta-analysis that aimed to understand potential intervention mechanisms, best practices, and suitable intervention components, as well as ways of measuring emotion regulation in youth [4]. We identified 21 studies, of which 9% (2/21) included some type of digital intervention. The results demonstrated a significant lack of technology-based interventions for youths and provided insights into the evidence base of existing psychological interventions and their impact on emotion dysregulation. Given the lack of any technology-based intervention for emotion regulation in our review, we had to rely on prior evidence that primarily focused on face-to-face interventions. A meta-regression suggested that changes in emotion dysregulation are associated with changes in psychopathology [4].

With respect to intervention components, the evidence was strongest for cognitive behavior therapy (CBT) approaches. We created an overview of the different intervention components (eg, psychoeducation, mindfulness, and attention bias modification), which subsequently formed the first basic tenets of the app (Table 3). CBT models and theories (eg, thought–feelings–behavior triangle) informed the content of a series of animated videos, which served as psychoeducational components. The animations also discussed strategies that are commonly used in CBT interventions to enhance emotion regulation, such as problem solving, cognitive restructuring, mindfulness, and relaxation. A more detailed description of the intervention components identified in the systematic review can be found in the study by Moltrecht et al [4].

In contrast to existing interventions and the limitations identified in the systematic review, the present app puts a greater focus on adaptive emotion regulation processes, as evidence from developmental studies suggested that the lack of adaptive emotion regulation in early childhood is associated with increased emotion dysregulation later in life [40].

**Table 3:** Design implications based on systematic review.

Findings	Design implications and goals
CBT^a^ interventions have the strongest evidence for face-to-face as well as digital interventions.	Integrate CBT concepts into the app, for example, psychoeducation about feelings, behavior, and thoughts
Interventions that improve emotion regulation also improve mental health.	Integrate exercises that enhance emotion regulation, for example, mindfulness
Emotion literacy, understanding, and differentiation are linked to better mental health.	Enhance children’s emotional literacy and understanding
Adaptive and positive emotion regulation are associated with less emotion dysregulation.	Include games that increase positive emotions and introduce adaptive emotion regulation strategies (eg, teach adaptive strategies)

^a^CBT: cognitive behavioral therapy.

### Design Implications of Stage 1

**Figure 3:**
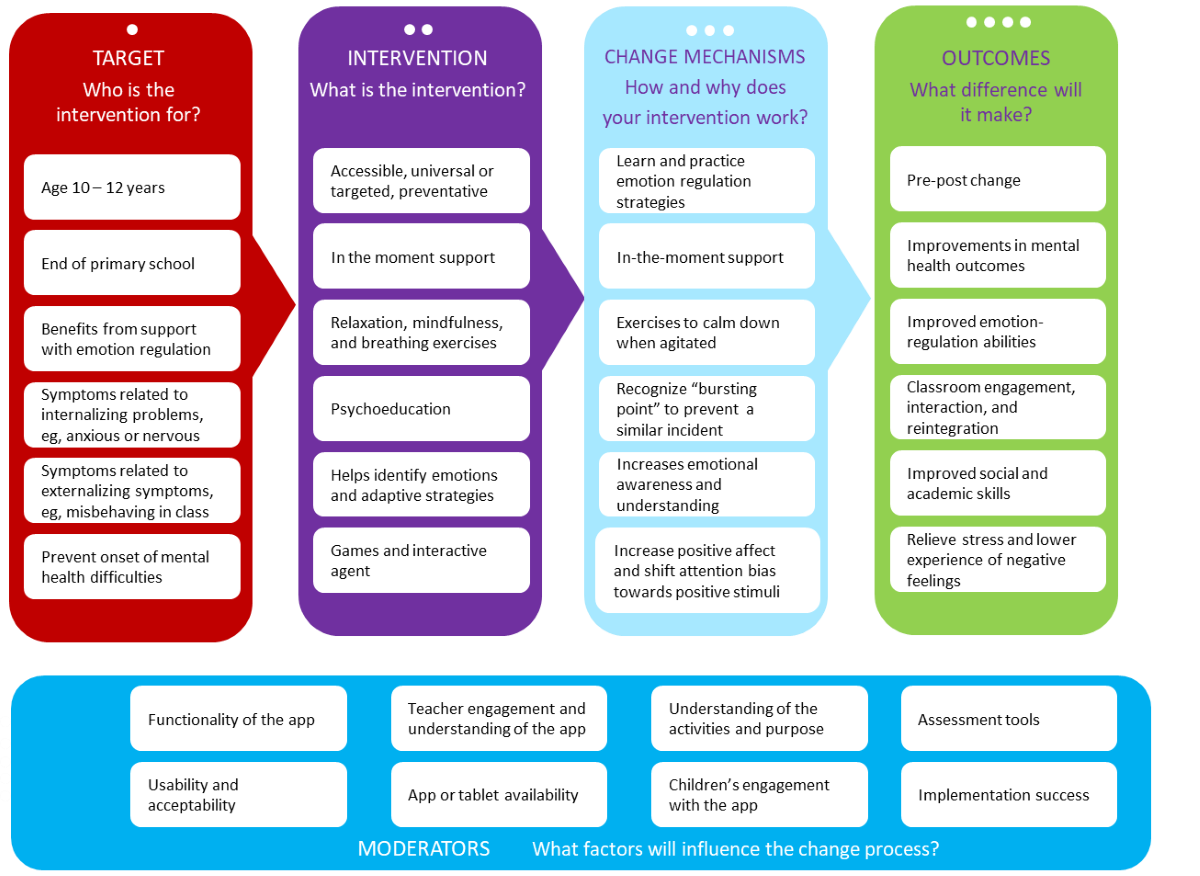
Stage 1 logic model of app intervention.

On the basis of the stage 1 findings, we outlined the different intervention components, change mechanisms, moderators, and outcomes in a logic model (Figure 3). The logic model was developed to clarify the conceptual and logical underpinnings of complex interventions used in child mental health services [34]. On the basis of our logic model and the outcomes of the PPI groups and school observations, initial wireframes were created by an app designer and developer. Furthermore, we decided on key criteria for the technology underlying the app (see the technology specifications in the following sections).

### Stage 2: Modeling and Design

#### Objectives

Stage 2 focused on involving children and other key stakeholders, including teachers, clinicians, researchers, the app developer, and designers, in the design and modeling process. This included the identification of key modules in the app, as well as the development of content and design of each module, such as psychoeducational content, games, and exercises.

#### Activities, Data Analysis, and Resulting Design Formulations

Stage 2 comprised a highly iterative process, combining the results of the PPI events (Table 2) and co-design and participatory workshops, followed by 3 testing sessions to make final design decisions and test the functionality and usability of the prototype. The combined use of PPI and user-centered design methods is highly recommended for the development of complex interventions [41]. Ethical approval to conduct these workshops was obtained from the University College London research ethics board (number 11701/001). We conducted 3 co-designs (N=15) and 3 participatory workshops (N=18) across 2 primary schools with 33 children (aged 10-12 years). Although specific demographic data, including the age and gender of the children, were not collected, the researcher team noticed that there was a slightly greater number of girls present in the workshops. Each workshop was conducted by the lead author and accompanied by a teacher.

The lead author took notes during each workshop and produced reflective notes afterward. Materials and notes produced during the workshops were coded manually and organized using mind-mapping exercises. Following this, a Microsoft Excel sheet was created to provide an overview of all items raised in the workshops. Each item was discussed with the app developer and designer to identify whether and how they could be integrated into the app. Each item in the Microsoft Excel sheet was color coded accordingly as follows: *green*=can be done, *orange*=might be possible or alternative suggestion to be tested in next workshop, and *red*=on hold or technology or design do not allow for this. On occasion, other stakeholders were involved, such as clinicians, to provide feedback on specific modules; their involvement is described in more detail in the respective sections.

### Co-design Workshops

The first co-design workshop introduced children to the project and their role as cocreators. After an icebreaker exercise, we explored what children knew about mental health and emotions, including what strategies they used in different emotion-eliciting situations. The first workshop did not involve specific wireframes, as the focus for this stage was to explore freely with children what activities and potential app features could help them regulate their feelings.

Children wrote down what feelings they knew (on sticky notes) and identified which feelings they found most difficult to regulate. Children reported that intense negative and positive emotions had a negative impact on their behavior in school (eg, “When I am super excited, I cannot concentrate” or “when I am angry, I don’t want to do stuff*.*”). In relation to this, children shared personal stories of situations that tended to elicit strong feelings in them either at school or at home.

On the basis of the children’s suggestions, we created a list of day-to-day strategies, which included strategies such as the following: *playing web-based games*, *listening to music*, *drawing and painting*, *watching something funny on YouTube*, *playing with my pet*, and physical activities such as *cycling* or *football with friends*. As a next step, we discussed the activities that could be supported through the app. The final list was then used to create overarching categories, which formed the core components of the app: (1) games to play, (2) something to relax, and (3) something to watch.

Going forward, we focused on these components as key modules while linking them to the stage 1 findings. For the games module, we created a list of possible games with the children. They frequently mentioned existing popular video games (eg, *Fortnite*) but also referred to other apps such as music making, coloring in, drawing in sand, and fast reaction games. In discussion with the app developer, each item was marked as *possible*, *alternative game*, or *not possible.* Complex games with multiple levels, requiring frequent updates, large amounts of data, or needing high resolution were discarded, as they conflicted with other design goals, such as (1) slowing down the app, (2) requiring too much data, or (3) not being suitable for a small mobile device. Owing to this, we had to exclude game ideas suggested by children, such as coloring in, music making, taking care and raising a pet, and a reaction game where the user smashes eggs by hitting them.

Simultaneously, the research team screened the literature to identify existing evidence for any of the games suggested or other games that have been developed in other contexts for this age group.

We asked the children what activities they found to be relaxing. Many suggested mindfulness and breathing exercises, which they had learned about at school. Others suggested *watching something on the web* or *listening to music.* This led to the idea of including music, or sound features, and encouraged us to make animated videos that could guide children with mindfulness and breathing exercises.

For the *watch* modules, we decided to develop a series of animated films. The storylines were inspired by children’s reports on their emotion-eliciting situations and the associated feelings, thoughts, and behaviors (eg, having a fight with a friend and not being able to concentrate in class). The stories were complemented with theories grounded in CBT (eg, the behavior–thoughts–feelings triangle) so that they could serve as a psychoeducational component. The scripts and screens were developed by a clinical psychologist and an animator who specializes in communicating mental health concepts to the public. Drafts of the films were reviewed by clinicians (N=7) who worked with children and in schools and a group of researchers specializing in child emotion regulation (N=5). Both provided feedback on the scripts and the visual presentation of the content and helped to ensure that they were in line with current evidence and guidelines. Any changes to the script or content were presented to children in subsequent workshops to ensure that they were age appropriate and that children could identify with it.

For the second and third workshops, wireframes and potential screen designs were printed on A3 paper based on the 3 core modules. Children were provided with pens, stickers, and sticky notes to add ideas for new features and review existing features. In contrast to the Druin [32] reports, but in line with recent observations by Jones et al [42], some children seemed to struggle with the creation of visual representations for potential app functions. It seemed as if they could not visualize how something that was drawn on paper could later be transferred to an app. As a result, some children were hesitant to draw their ideas and preferred to describe them. Therefore, we decided to build a basic but high-tech prototype for subsequent workshops, which seemed to make it easier for children to provide suggestions for existing and new app features.

### Participatory Workshops

We asked the children to provide feedback on the high-tech prototype that we developed based on the outcomes of the co-design workshops. In comparison to the low-tech paper prototypes, the high-tech prototype made it significantly easier for children to find their role in the process and provide suggestions for and against potential app features.

During the participatory workshops (N=18), children raised the need for a feature that provided in-the-moment support. They reported that it was difficult to remember helpful strategies when they experienced strong feelings. Following this, a *help button* was added, which children could press when they were experiencing strong emotions and could not remember the tools or strategies available to them.

Moreover, children suggested that it would help them if they could tell the app how they felt, and it told them in return what they could do about their feelings (“Can I tell it how I feel and it tells me what to do?”). This possibility was first explored through a chatbot function, whereby children could tap on an animated agent on the home screen to open a chat window. When we tested this feature in subsequent workshops, it became evident that some children thought that they were speaking to an actual person (ie, “Who is on the other side?”). Therefore, we decided against the chatbot function for this age group, as it involved potential risks, for instance, if a child needed urgent help and tried to access it through the chatbot. Although the chatbot function presents an exciting opportunity for engaging children with the app, developing it further was beyond the scope of this research. Hence, we decided to replace the chatbot with a *check-in function*. With this feature, children could select a feeling from a list to indicate their emotional state, and in return, the app would provide suggestions on what to do. This feature was considered a safer alternative by clinicians and researchers and required less complex functionality and development time (see *The Check-in Function* below).

For the check-in function, an initial list of 12 feelings was created based on the most common feelings that children reported in the first round of the co-design workshops. We designed a set of images, each representing 1 of the 12 feelings. We tested the validity by showing children the images without a description and letting them rate what emotions were represented. On the basis of the children’s feedback, the images were further adjusted. Children also highlighted important emotions that were missing; therefore, the list was extended. The final list aimed to reflect a full range of feelings, ranging from emotions with positive or neutral valence to negative valence, as well as different levels of arousal. For instance, *feeling excited* represents an emotion of positive valence and high arousal, whereas *feeling grateful* is a state of positive emotional valence but low arousal.

Consequently, the functionality of the animated agent was reduced to two main functions: (1) *tell me something*, which activated a random selection of jokes or funny facts that were expected to increase the level of engagement and perceived level of interaction with the app, and (2) the *check-in* function, as presented above.

A summary of all items raised in the workshops and the resulting changes to the app are presented in Table 4.

**Table 4:** Observations and design implications based on participatory design workshops.

Observations and feedback	Implications, solutions, and actions
Games to play and feel happy	We created a list of games to discuss with the app developer. Solutions and suggestions were tested and further adjusted with children in the next workshop.
Children suggested breathing and mindfulness exercises, which they knew from school.	We developed animated videos to guide them through exercises.
Children would like a feature to create music. Children listen to music to relax.	Music making feature conflicted with the usability of the app. We added music and sounds to the relax module.
Children frequently reported watching videos as a way of calming down, relaxing, being happy, and distracting themselves.	We created video content for watch modules.
“Can I tell it how I feel and it tells me what to do?” indicated that children would like some guidance and support in difficult situations and with specific feelings.	We explored the chatbot function, which was then replaced with the check-in function.
Children requested in-the-moment support when feelings were too intense.	We added an easy-to-reach help button to the home screen of the app. Once pressed, a stop and breathe sign covered the whole screen, which is followed by a guided breathing exercise.
Children thought that they were talking with a real person in chat.	We replaced the chatbot with a check-in function.
Children liked to interact with the digital agent and wanted more of that.	We kept the digital agent and added interactive features.
Children reported watching and listening to funny things to feel better.	We asked children for jokes and fun facts and added these to the animated agent, which was in line with our goal of increasing opportunities for interaction.
Speech bubbles of agent too fast	We increased the length of time of the speech bubbles.
The onboarding process required more colors and options	We added background colors and a selection of different color themes.
Explainer on how to use the app	We added stars to highlight different functions in the app for first-time users. This was discarded as it was too complex to adjust the position of the stars on the screen for different screen sizes. We added a short explainer video as part of the onboarding process.

### Prototype Testing

The prototype was tested in another primary school with 15 children across 3 workshops. During these workshops, broad design features, such as the flow of screens, as well as more detailed design questions regarding language and use of colors, were discussed. The schools provided tablets, which allowed us to test the functionality of the app across different devices and the school’s technology infrastructure (eg, access to Wi-Fi and digital safety policies of the school). The teacher was present at each workshop.

At the beginning of the workshops, children were informed about the purpose of the app but were not given any instructions on how to use the app. This allowed us to observe whether the current design was intuitive enough for children to use it without much explanation. Children were encouraged to speak out loud their thoughts while navigating through the app. A researcher observed the children and took written notes about the ways in which they explored the app to identify pitfalls, popular items, technical difficulties, and features that they did not discover on their own. Following this, children received an in-depth introduction and were asked to be technology detectives who helped us find any glitches and errors. All children were encouraged to provide honest feedback and suggestions concerning the usability of the app and how it could be improved.

BM took part in each workshop, asked follow-up questions, and took notes. After each workshop, BM made reflective notes and asked teachers about their observations. The data were organized in a Microsoft Excel spreadsheet according to specific app functions. The spreadsheet was used to discuss each item with the app developer and designer, who subsequently adjusted the app. A summary of the items raised and adjustments made is provided in Table 5.

**Table 5:** Observations and implications following prototype testing.

Observations and feedback	Design implications and actions
Difficulties with certain functions dependent on different uses of browsers	Test the web-based app across different browsers and devices
The screen and design did not adjust correctly for devices of different sizes.	Test with different screens and devices
Animated videos were sometimes slow.	Improve video quality Make them available offline
Add personalized tools to the tools list Differentiate between in-class and out-of-class tools list, as some tools, for example, go outside, were not suitable for the classroom context	Children can mark favorite tools in the list. Two tabs for different contexts
Problems with log-in. Children either did not have an email address or forgot log-in details.	Add different log-ins for quick classroom access and use at home
Music did not stop when leaving the module	App developer checks stop and pause functions, and music and videos automatically pause when leaving a module
Some of the games did not start.	App developer adjusts underlying technology

## Results

### Intervention Design and Evidence Base: Technical Specifications

The intervention was developed as a responsive web-based app to increase the accessibility of the app, as it allows users to access it from different mobile devices, desktop computers, and smartboards. Although it works across multiple platforms, it was optimized for tablets, as children are more likely to have access to tablets at school and at home [43].

The app is delivered through a browser, meaning over-the-wire updates could be pushed out instantly, and the app uses advanced HTML5, cascading style sheets level 3, and JavaScript (ECMAScript 6) techniques to render a smooth and performant UX. The underlying development platform used was Meteor.js, a full-stack Node.js application development framework, hosted on a resilient Amazon Web Services Elastic Compute Cloud-2 instance with a MongoDB database hosted via MongoDB Atlas. The app only requires an internet connection when users access it for the first time, after which it can be saved to the home screen. This feature was chosen to mitigate risks that the intervention could not be accessed in the case of reduced or limited Wi-Fi.

The app offers two different types of log-ins: a guest log-in and a registered account log-in. The guest-log-in serves the following purposes: (1) new users can explore the app without having to register, (2) children without an email address can use the app, and (3) it allows for easy and fast access. The second log-in allows users to set up a personal account registered through an email address and password. The app only remembers personalized features (eg, design features) when users access it through their personal account. The app does not store any individual user data and adheres to existing general data protection regulations.

### Intervention Description

**Figure 4:**
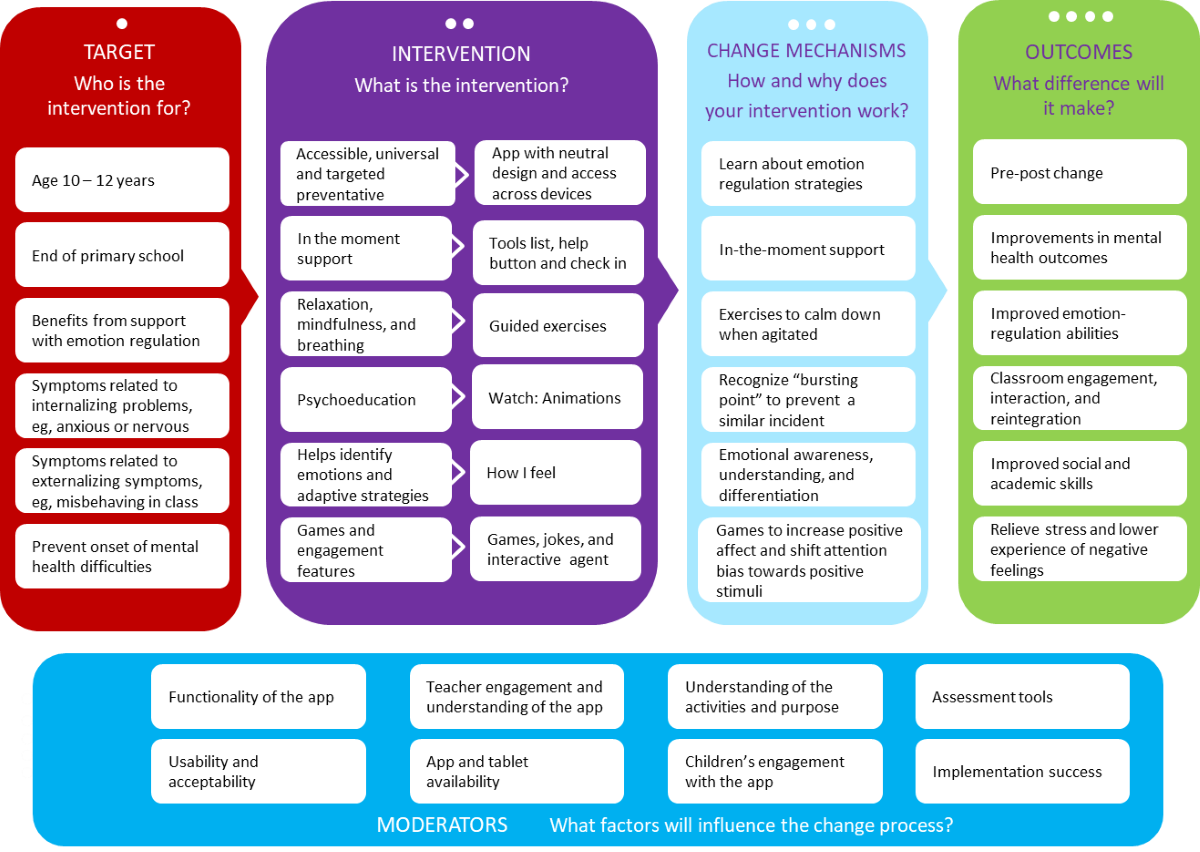
Finalized logic model for app intervention.

The latest version of the intervention includes four modules: play, relax, watch, and tools. The different modules provide users with opportunities to learn, practice, and develop their emotion regulation skills. The content is presented through audio tracks, images, animated films, and games. On the basis of the activities and findings outlined above, we adjusted the logic model further (Figure 4).

### Onboarding Process and Home Screen

#### Overview

**Figure 5:**
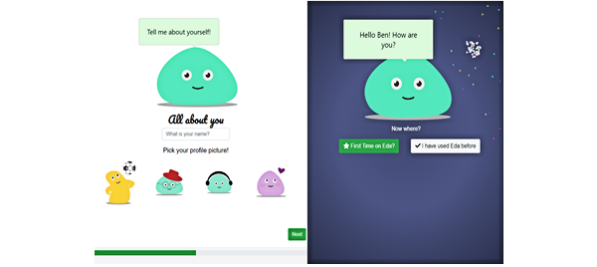
Onboarding screens of the app.

**Figure 6:**
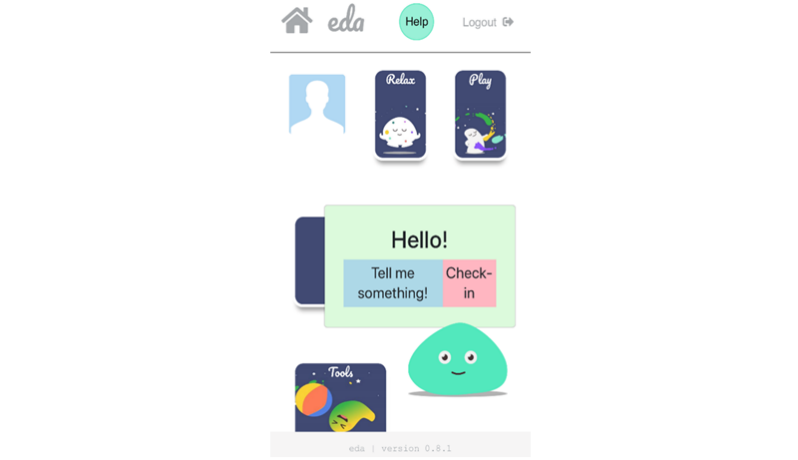
Home screen of the app with 4 main modules and the digital agent.

First-time users go through an onboarding process before they reach the home screen of the app. During this process, they learn about the purpose of the app, provide account details (eg, username), and select a preferred color scheme and profile picture (Figure 5). After the onboarding process, the user enters the home screen, where Eda, an animated digital agent, greets them with their chosen username. Eda encourages them to explore the app or tap on its body to open the *check-in* or *tell me something* functions. The latter activates a random selection of jokes or funny facts to increase the level of engagement with the app (Figure 6).

#### The Digital Agent

We aimed to design Eda as a gender-neutral, animated agent who accompanies the user through the different modules in the app. This feature was added based on the children’s requests to have someone to turn to in situations where strong emotions are experienced. In addition, research has shown that the use of animated agents can facilitate the experience of having a personal relationship, which in turn increases long-term engagement with a digital intervention [44]. The digital agent was designed as a moving (or *wobbling*) blob with big, blinking eyes to add a human feel to it, which is hoped to enhance a feeling of connectedness and engagement in the user [45].

#### The Check-in Function

**Figure 7:**
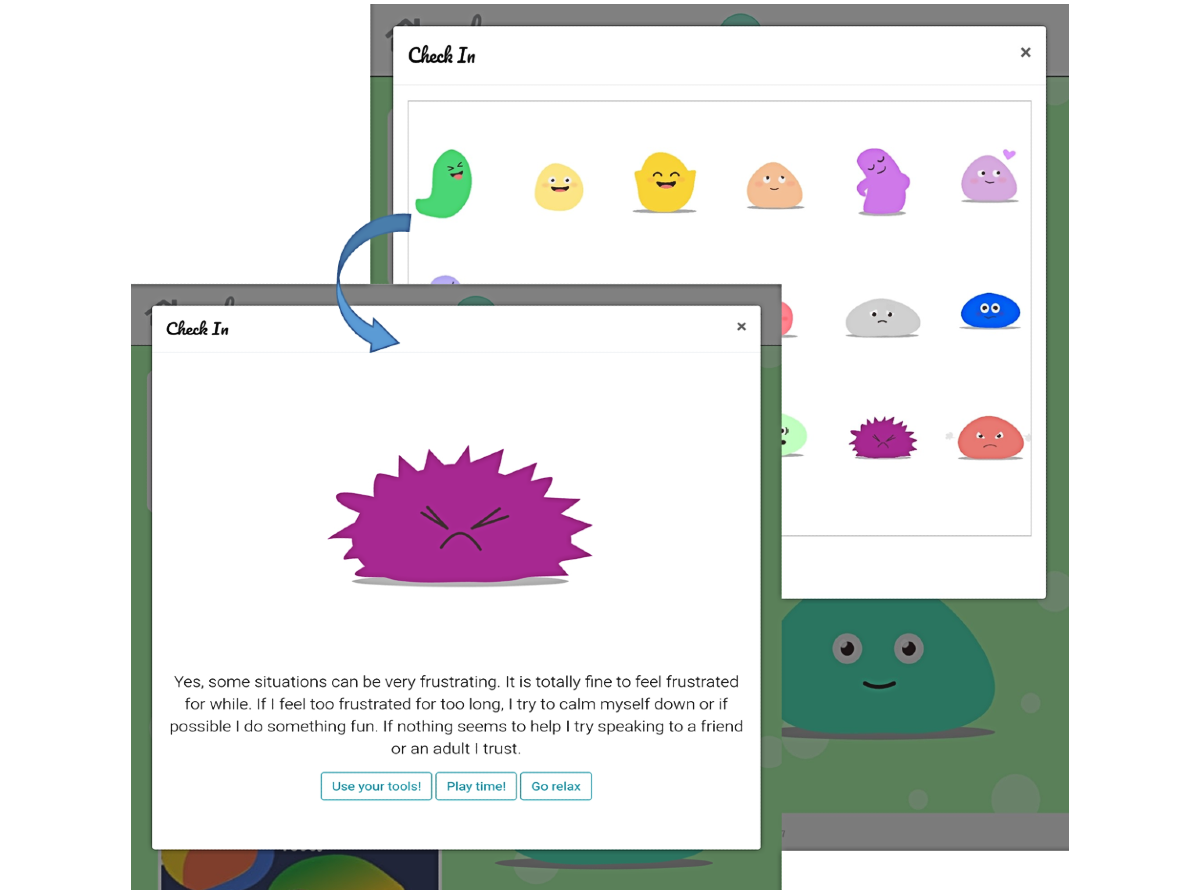
Check-in function in the app.

The *check-in* function (Figure 7) displays a set of 18 different feelings to the user. When the user selects a specific feeling, a new window opens up that provides more information about the chosen feeling and provides suggestions regarding potentially helpful emotion regulation strategies. Where appropriate, cross-links to other modules in the app are provided (eg, relax) so that the user has the opportunity to immediately apply or practice these strategies. This approach is in line with past research that has structured emotions along the 2 dimensions of arousal (high vs low) and valence (positive vs negative) [46]. It has been suggested that internalizing symptoms are associated with the primary experience of low-arousal emotions, whereas externalizing symptoms are linked to high-arousal emotions [47]. We believe that this function not only meets the children’s initial requests but can also help them expand their emotional literacy and emotion differentiation skills, which has been linked to better mental health and is therefore in line with the purpose of the present app [48-50].

#### Education and Practice Modules

**Figure 8:**
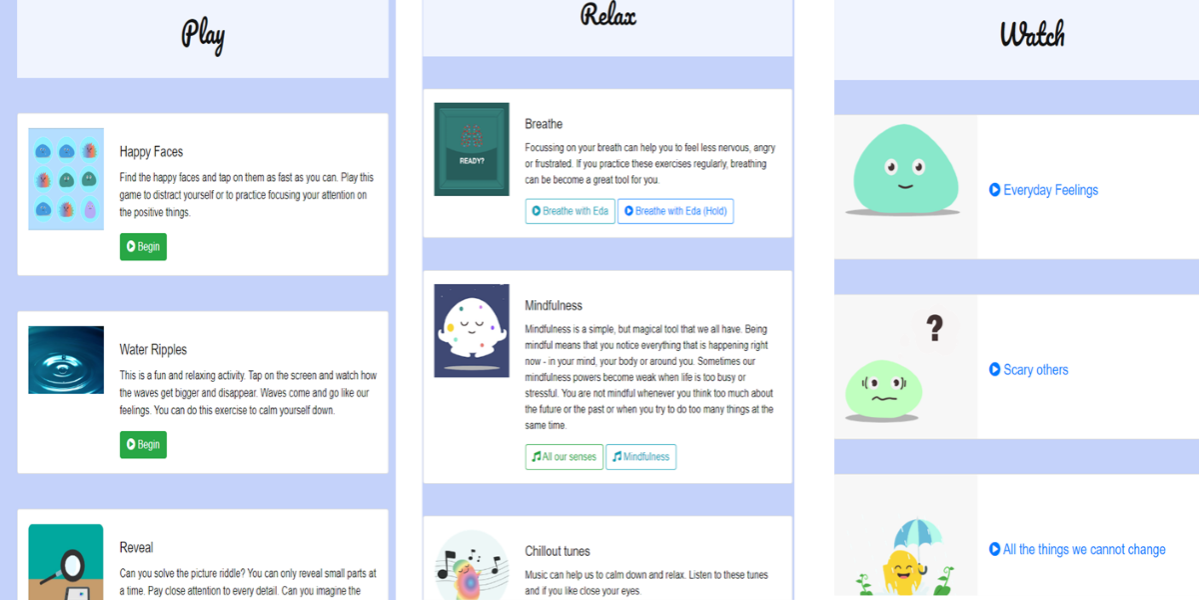
Content of Play, Relax, and Watch modules.

Users can access 1 of the 4 main modules (Figure 8) manually via the home screen or by selecting an emotion in the check-in function, which subsequently forwards the user to one of the practice modules.

##### Play

**Figure 9:**
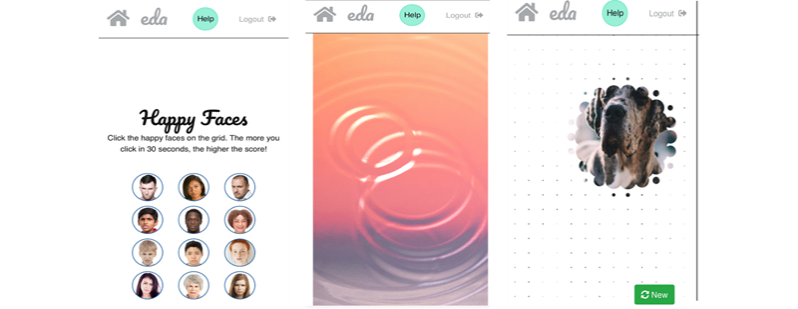
Overview of games, including Happy Faces, Water Ripples, and Reveal.

This module contains 3 games (Figure 9). In the first game, *Happy Faces*, the user must identify 1 happy face among 12 neutral or angry faces. This design was chosen because research shows that search tasks such as these can result in an attention bias shift toward positive stimuli, which in turn increases the likelihood of experiencing more positive emotions [51]. During one of the workshops, some children suggested that the game should have a second level of increased difficulty by animating the faces so that they move over the screen like balloons. In discussions with the app developer, this specific feature was considered too complex for the present version of the app but will be further explored in the future.

The second game, *Water Ripples*, presents a colorful picture with an animated water surface. By tapping the screen, the water animation creates circular waves that slowly expand to the sides of the screen. The design evolved from the co-design workshops, where children reported that drawing in sand or water drops had a calming effect on them. Furthermore, it resembles a commonly used mindfulness exercise, in which individuals imagine their emotions as waves that come and go [52].

The third game, *Reveal*, shows a colorful picture that is covered by a white layer. By touching the layer with a finger, parts of the white layer disappear and reveal sections of the underlying picture. The user is encouraged to guess the theme or object of the underlying picture. Although there was no specific psychological theory to guide the design of this game, especially in the context of a mental health intervention, research has shown that games such as the ones chosen here foster engagement [53]. Furthermore, games have been shown to increase positive affect and well-being, although more research is needed to identify which specific aspects initiate the change and whether or how this might differ for different users [54,55]. When testing this game with the children for the first time, it became evident that they (1) wanted to know whether they identified the correct underlying picture and (2) that adding a point or reward system for correct answers could further increase their engagement with this game.

##### Relax

**Figure 10:**
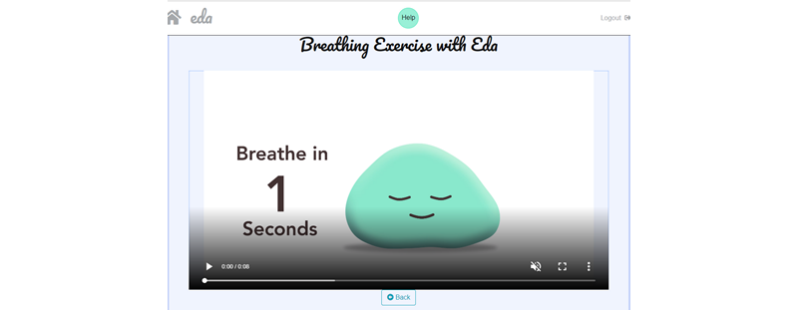
Screenshot of guided breathing animation.

The relax module was inspired by the school observations and reports from children during the workshops, which indicated that most schools already used breathing and mindfulness methods; hence, many children were already familiar with relaxation exercises. In line with this, increasing evidence shows that mindfulness interventions enhance emotion regulation and exert positive effects on mental health and well-being [56,57]. The relax module contains 3 sections that encourage the user to actively engage in some type of relaxation or mindfulness exercise. The user can choose from video animated breathing exercises (Figure 10), audio-guided mindfulness exercises, and a selection of calming sounds (eg, guitar or rain). The decision to include sounds or relaxing music was based on the children’s suggestions in the workshop, as well as classroom observations, where teachers used music to keep children concentrated during a task.

##### Tools List

**Figure 11:**
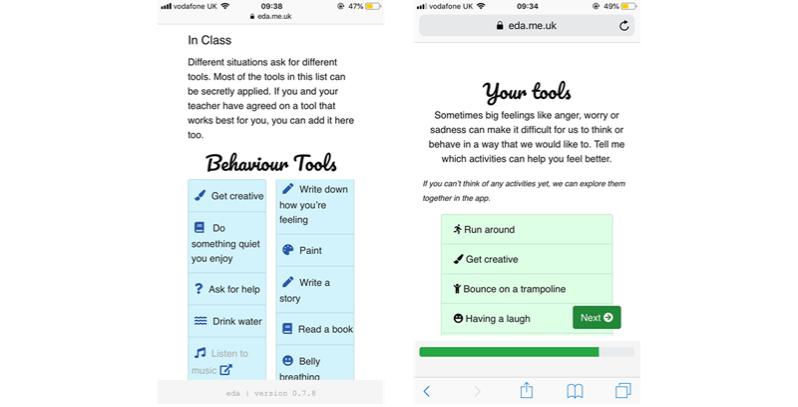
Tools list feature showing different tools for different contexts and personalized tools.

The tools module evolved from conversations with children who indicated that they used different methods to regulate their emotions; some of these methods were developed with the help of a teacher. Thus, the tools module comprises a list of behavioral and cognitive strategies that are expected to help with intense emotions (Figure 11). The list is divided into a general tools list that can be referred to outside of the classroom (eg, doing something fun and getting support from a friend) and a specific list suitable for the classroom (eg, going to the quiet zone).

With respect to existing evidence demonstrating that the lack of and limited access to appropriate emotion regulation strategies contributes to mental health difficulties, it was expected that giving users easy access to these tools would positively influence their emotion regulation abilities [58]. Furthermore, research has shown that early school years represent a crucial time for children to expand their repertoire of emotion regulation strategies, including cognitive and behavioral strategies [59]; hence, it was assumed that the tools list could positively support this development.

##### Watch

This module contains animated psychoeducational films to improve users’ understanding of emotions, emotion regulation strategies, and how thoughts and behaviors influence emotional experiences. This is achieved by explaining commonly applied CBT principles in simple terms and by introducing some of the more complex emotion regulation strategies, such as cognitively restructuring one’s thoughts (ie, cognitive reappraisal) or mindfulness [60,61]. Research has shown that CBT-based interventions successfully improve a variety of psychopathological symptoms, even if delivered through technology-based platforms [15]. Furthermore, the results of the systematic review demonstrated that CBT-based interventions were effective in improving emotion regulation difficulties in youths.

#### Help Function

This function was included based on the children’s requests to have more in-the-moment support when they experience high levels of negative emotions, which can prevent them from engaging in adaptive decision-making.

Therefore, by clicking on the help button, a series of emotion regulation methods are presented to the user (ie, stop what you are doing, count to 3, and breathe), who is instructed to follow them until the initial emotional reaction decreases to allow for more adaptive actions. This functionality is also in line with research indicating that the duration of an emotional experience is influenced by the type of emotion regulation strategy used [62]. It was expected that the help function would support children to distract themselves from emotion-eliciting stimuli. Distraction is an emotion regulation strategy that has been shown to quickly decrease the levels of negative emotions [63,64]. Similarly, the use of distraction strategies to regulate intense emotions is a substantial part of dialectic behavioral therapy, which has been shown to effectively support individuals with severe emotion regulation problems [65,66].

## Discussion

### Principal Findings

Mobile apps for children represent a promising pathway for providing effective mental health support; however, there is a significant lack of mental health apps for this age group (ages 10-12 years) [7,9]. Only recently, the self-management intervention ReZone was developed for children (aged 10-15 years) with the aim of reducing internalizing and externalizing symptoms [67]. Early findings suggest that the app was perceived as helpful by pupils; however, findings from a proposed randomized controlled trial have not yet been published. In addition, Hides et al [68] developed a new music app to enhance emotion regulation in adolescents, and their initial findings with young people (N=169; aged 16-25 years) suggested that the app could potentially enhance emotion regulation; however, further testing is required to determine its effectiveness.

As highlighted above, for many mental health apps for children, information on the design and testing process is not available [7]; hence, we address this gap by describing and sharing our development and design process as we continue to develop the app further.

### Strength, Limitations, and Lessons Learned

A significant strength of the present development process is the inclusion of children and young people at every stage. Owing to existing collaborations, we were able to involve young advisors (aged 12-19 years) in the early stages of our project, which had numerous benefits. However, the inclusion of slightly older participants in the PPI events may have also contributed to some of the issues we experienced in the workshops with younger children. We believe that the work with young advisors was very valuable but want to highlight that the involvement of the target users should be a priority when designing new digital interventions.

By combining methodologies from different fields, we adopted a highly interdisciplinary approach, the lack of which has been highlighted as a significant limitation in existing digital mental health interventions. We hope that in doing so, we increased the potential for sufficient user engagement while also providing a sound evidence base for the content of the intervention [12,15]. Despite our best efforts, it was not possible to have all the different experts in one room for the workshops. This can be particularly difficult in research that includes vulnerable populations, where additional safeguarding regulations are in place. Such access constraints affecting the work of HCI researchers and designers with vulnerable groups have been highlighted before [69]. In our project, the lead researcher was already trained to work with children and had easier access to the target group. To facilitate our interdisciplinary approach, BM undertook additional training to familiarize herself with the methods from the different disciplines and consulted experts from other fields before and after each activity. Throughout the development process, the lead author served as a linking point for all stakeholders and tried to gain and share everyone’s views and opinions. Although in an ideal scenario, experts from different fields would be conducting the workshops together, we believe that we took the best possible approach by training the lead researchers in interdisciplinary methods and having regular consultations with experts from the respective fields.

Although the inclusion of various experts and stakeholders in the process is a significant strength of our development framework, we believe that this aspect could be further improved by developing a decision-making tool with all stakeholders beforehand. Such a tool could be consulted whenever contradicting design goals from different stakeholders need to be addressed. Our team did not develop such a tool, and final decisions were made by the research team, which may have resulted in unwanted biases.

The collaborative approach with schools had various benefits, as it ensured regular access to the user group and helped us identify context-specific design goals at an early stage. Furthermore, the research team was able to conduct all the design workshops within the school context, as recommended in the cooperative inquiry framework [32]. However, during the workshop activities, the research team noticed that children who were reported to show the most emotional and behavioral difficulties at school were also less engaged in the workshop. The research team had the impression that some of these vulnerable children may have engaged more in a different context. Going forward, we would suggest speaking to these children outside of the school context or choosing different workshop activities so that all voices can be taken into account. Furthermore, in most cases, the teachers decided which children would join the workshop activities. This could have caused an unwanted bias, as previous research suggests that adults were less likely to choose children with certain characteristics (ie, less sociable, externalizing symptoms, and lower academic competencies) [70]. In relation to that, it should be noted that the research team did not collect specific demographic or other sensitive category data, which could be useful in interpreting current but also future use data. Collecting data on participant characteristics can provide insights into other mental health risk factors (eg, existing mental health problems, living status, and ethnicity), which can help in comparisons among different user groups that may be of the same age but have different mental health profiles. Therefore, we suggest that future research should collect relevant participant data during the early design stages.

Teachers contributed tremendously with their views and expertise. However, they had very limited time available, and their role as intervention deliverers has not yet been fully addressed at this stage. We suggest conducting more classroom observations with a specific focus on teachers’ roles and needs to adjust the app accordingly. This could also be explored as part of an exploratory feasibility trial, which we suggest as a next step to develop and evaluate the present app further. We suggest a series of exploratory feasibility trials to uncover and iron out the remaining technology and design issues. Moreover, with respect to one of the greatest limitations of today’s digital mental health interventions [12], we suggest that the next stages need to focus primarily on usability, engagement, and implementation of the present app before any effectiveness testing.

Throughout the development process, we noticed a tension between finding the right balance between guaranteeing an adequate evidence base for each feature of the intervention and leaving enough room for creativity and innovation of new features. We strongly agree that evidence-based and evidence-informed concepts are of significant importance; however, there seems to be a common misconception that one can only achieve *evidence-based innovation* by *transforming* evidence-based nondigital interventions into digital ones [17]. We would like to encourage the field to free itself from this notion as it can significantly hinder us from reaching the actual potential of digital mental health interventions [71]. In relation to this, we would like to refer the readers to the findings of our exploratory trial [29], where we discuss promising future directions for the present app.

### Conclusions

Digital interventions that target transdiagnostic mechanisms such as emotion regulation have the potential to support young people’s mental health on a wider scale, regardless of the level or type of symptoms that they experience. Currently, little guidance exists on how to develop such transdiagnostic digital interventions for children. We highlight the existing limitations in the field and present a new approach to address them in this project. By developing a new interdisciplinary development framework, we were able to incorporate methods from different fields. Although more research is needed to evaluate and further improve this app, we hope that sharing our insights and lessons learned in this paper will be a helpful guide to others.

## Abbreviations

CBT: cognitive behavior therapy
HCI: human–computer interaction
PPI: patient and public involvement
UX: user experience
